# Valorization of Olive Mill Wastewater by Selective Sequential Fermentation

**DOI:** 10.3390/foods14132170

**Published:** 2025-06-21

**Authors:** Lara Signorello, Mattia Pia Arena, Marcello Brugnoli, Flora V. Romeo, Maria Gullo

**Affiliations:** 1 Department of Life Sciences, University of Modena and Reggio Emilia, 42122 Reggio Emilia, Italy; lara.signorello@unimore.it (L.S.); marcello.brugnoli@unimore.it (M.B.); maria.gullo@unimore.it (M.G.); 2 Council for Agricultural Research and Economics (CREA)-Research Center for Olive, Fruit and Citrus Crops, 95024 Acireale, Italy; floravaleria.romeo@crea.gov.it

**Keywords:** olive mill wastewater, alcoholic fermentation, acetic acid bacteria, acetic beverages, by-products

## Abstract

Olive mill wastewater is a by-product of olive oil extraction, characterized by a high concentration of organic matter, which presents a significant environmental challenge if not properly managed. This study was aimed at valorizing olive mill wastewater through selective fermentations to produce acetic beverages with low or no alcohol content. Olive mill wastewaters at three different dilutions (100%, 75% and 50%) were inoculated with *Saccharomyces cerevisiae* UMCC 855 for alcoholic fermentation. The resulting alcoholic product, with 75% olive mill wastewater, was then used as a substrate for acetic acid fermentation by *Acetobacter pasteurianus* UMCC 1754, employing both static and submerged acetification systems. The results showed that, at the end of the static acetification process, no residual ethanol was detected and that high concentrations of acetic and gluconic acid (46.85 and 44.87 g/L, respectively) were observed. In the submerged fermentation system, the final ethanol concentration was 24.74 g/L; the produced organic acids content reached 31.63 g/L of acetic acid and 39.90 g/L of gluconic acid. Furthermore, chemical analyses revealed that fermentation enhanced the antioxidant activity of olive mill wastewater. These results suggest promising insights for the valorization of olive mill wastewater.

## 1. Introduction

*Olea europaea* L., an evergreen tree belonging to the *Oleaceae* family, is cultivated mainly in the countries of the Mediterranean area, including Italy, Spain, Tunisia, and Greece. Drupes can be consumed as natural fermented table olives or subjected to several transformation processes. Olive oil is the most important derived product and represents a strategic food commodity for the Mediterranean area. To obtain olive oil, different extraction methods are used, i.e., pressure, centrifugation and percolation by selective filtration [1]. However, olive processing for obtaining oil has a high environmental impact due to the large amount of water used and the resulting olive mill wastewater (OMWW) production.

The chemical composition of OMWW is influenced by many factors, including the olive variety and its maturity, climatic conditions, and the oil extraction process used [2]. Generally, OMWW is mainly constituted by sugars, including fructose, mannose, glucose and sucrose, phytotoxic substances, and microbially inhibitory compounds such as phenols and long-chain fatty acids.

The high concentration of phenolic compounds, the high organic load, and the chemical oxygen demand are responsible for the phytotoxic effects of OMWW on the environment. These characteristics have historically limited its direct disposal or reuse [3,4]. Nevertheless, due to its rich organic and bioactive content, OMWW has attracted growing interest for potential applications as a source of valuable compounds. In response, several physical, chemical, biological, and combined treatment technologies have been developed not only to reduce its environmental impact but also to explore sustainable reuse strategies [5].

In recent decades, the attention of governments and public opinion towards environmental protection and environmental sustainability have increasingly focused on reducing the negative impact that human activities have on the environment. Nowadays, the management and reuse of OMWW in agricultural soils are not regulated by European Union (EU) legislation. Consequently, it is necessary to develop technologies to reuse OMWW to avoid their discharge into natural waterways [6]. Circular economy principles are driving the production and consumption models that boost the reuse and recycling of materials and by-products. These include: anaerobic treatment focused on the production of biogas for energy purposes and partially stabilized organic matter for soil amendment [7]; solid fermentation by microorganisms to obtain feed, enzymes, and fuel production [8]; composting with other agricultural wastes to gain a final product with good humification [9]; the extraction of valuable products, such as pectin [10], for the food industry; and tyrosol, hydroxytyrosol, caffeic acid, oleocanthal and oleuropein [11] for possible use in the pharmaceutical industry. Other studies on OMWW reuse include its use as an absorbent for heavy metals in the treatment of aqueous solutions to reduce, for example, biodegradation and leaching [12].

However, there are several limiting factors such as the investment costs for depollution treatment technologies and the high level of technological know-how required [13].

In the frame of upcycling by-products, such as OMWW, from the food industries, a valuable resource is represented by the inclusion of these by-products in the formulation of functional foods, offering the potential to create a wide range of products with significant health benefits [14]. Indeed, food by-products can be effectively incorporated into various food products, with plant-based fermented beverages standing out as a particularly promising application. Fermented beverages from date palm fruits, kiwifruit, prickly pear, pomegranate juices, and non-conventional edible plants are increasingly popular thanks to their health-promoting properties [15]. As fermented beverages are perceived as a healthier alternative to conventional soft drinks, there is a growing demand for alternative raw materials suitable for fermentation [16]. Furthermore, leveraging by-products for beverage production aligns with zero-waste principles and can help to reduce manufacturing costs, making it an appealing and sustainable approach. Fermentation offers an effective method for transforming these by-products into value-added beverages.

On this basis, this work aimed to valorize OMWW by developing acetic beverages (2–4% *w*/*v* of acetic acid) with low or no alcohol content through pilot-scale static and submerged fermentation regimes. First, diluted and undiluted OMWW were used to produce alcoholic products. The alcoholic fermentation was carried out by the *S. cerevisiae* UMCC 855 strain, previously tested in different conditions [17,18]. Moreover, the suitability of *S. cerevisiae* strains to ferment OMWW has been reported by several authors [19,20,21]. Consequently, the best alcoholic product was used as substrate for the acetification. The acetic acid bacteria (AAB) strain used for the acetic acid fermentation phase was chosen based on a selection within strains of *Acetobacter pasteurianus* and *Komagataeibater europaeus* species due to their relevance in transforming ethanol into acetic acid, both in static and submerged fermentation regimes [22]. Fermentation processes were implemented following the general practices used for vinegar-making, which included both static fermentation and submerged regimes.

Overall, the study provides the basis for the industrial scale-up of bioprocesses for the valorization of OMWW through the production of acetic beverages.

## 2. Materials and Methods

### 2.1. OMWW Samples

The OMWW samples were collected in November 2023 from a three-phase olive mill plant located in Catania (Sicily, Italy). Prior to utilization, OMWW samples were centrifuged three times at 6000 rpm for 15 min at 25 °C to remove solid parts. The liquid fraction of OMWW was filtered and cold-sterilized in a single step using a vacuum filtration system equipped with a 0.2 µm PES membrane (Nalgene Rapid-Flow, Thermo Fisher, Waltham, MA, USA), then stored at −20 °C until use. This approach allowed for the removal of microbial contaminants while preserving the chemical integrity of the OMWW.

### 2.2. Bacterial Strains and Culture Conditions

In this study, yeast and AAB strains were obtained from UMCC (Unimore Microbial Culture Collection, Reggio Emilia, Italy) and DSMZ (Deutsche Sammlung von Mikroorganismen und Zellkulturen, Braunschweig, Germany) (Table 1). Strains were recovered from −80 °C storage conditions in YPD broth (yeast extract 10.0 g/L, peptone 20.0 g/L, glucose 20.0 g/L,) and GY broth (glucose 50.0 g/L, and yeast extract 10.0 g/L), respectively, for yeast and AAB. The yeast strain was incubated at 28 °C for 1 day; AAB strains were grown at 28 °C for 4 days.

### 2.3. General Experimental Workflow

**Figure 1:**
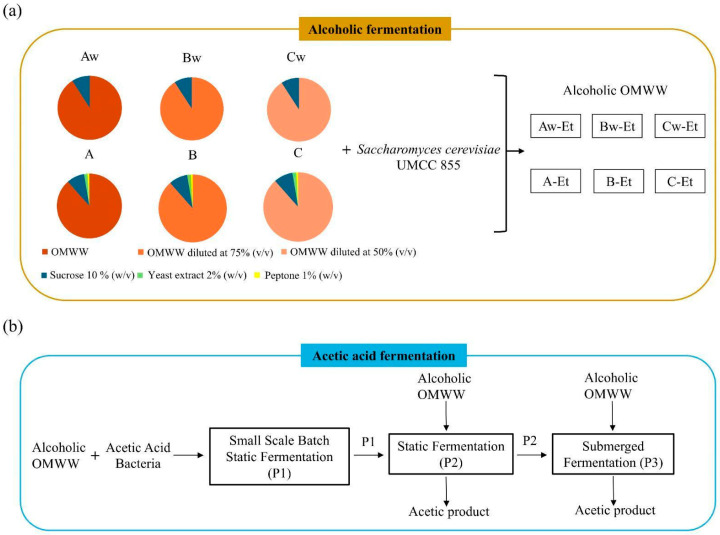
Experimental workflow: (**a**) undiluted OMWW (A) and OMWW diluted with distilled water to 75% (*v*/*v*) (B) and 50% (*v*/*v*) (C) were supplemented with 10% (*w*/*v*) of sucrose (Aw, Bw, Cw) or enriched with 10% (*w*/*v*) of sucrose, 2% (*w*/*v*) of yeast extract, and 1% (*w*/*v*) of bacteriological peptone as nitrogen sources (A, B, C). Samples underwent alcoholic fermentation with *S. cerevisiae* UMCC 855, resulting in alcoholic OMWW (e.g., Aw-Et, Bw-Et, Cw-Et, A-Et, B-Et, C-Et); and (**b**) alcoholic OMWW was then used for acetic acid fermentation by acetic acid bacteria, conducted in static (P1 and P2) and submerged (P3) fermentation systems, respectively.

The present study explored a fermentative approach for the valorization of OMWW, aimed at producing acetic beverages with low or no alcohol content. Figure 1 illustrates the general experimental workflow. Diluted (75% and 50% *v*/*v*) and undiluted OMWW samples were enriched with sucrose (10% *w*/*v*) or sucrose (10% *w*/*v*) with nitrogen sources (2% *w*/*v* of yeast extract, and 1% *w*/*v* of bacteriological peptone) and then subjected to alcoholic fermentation. Alcoholic fermentation was carried out with the *S. cerevisiae* UMCC 855 yeast strain, previously studied for its oenological aptitude [18]. The alcoholic product (B-Et) was used as substrate for the acetic fermentation, with an initial small-scale batch static fermentation (Phase 1, P1), followed by two systems: static fermentation (Phase 2, P2); and submerged fermentation (Phase 3, P3). To set the acetic acid fermentation of alcoholic OMWW, AAB strains of *A. pasteurianus* and *K. europaeus* species were screened. The resulting acetic products from static and submerged fermentation were labelled, as reported in Table 2.

### 2.4. Alcoholic Fermentation

OMWW samples Aw, Bw, Cw, A, B and C, were subjected to alcoholic fermentation. To obtain an inoculum of 10^8^ CFU/mL, 5 mL of UMCC 855 yeast culture was centrifuged at 6000 rpm for 10 min at room temperature and the pellet was resuspended into 50 mL of OMWW samples. After 2 days, the scaling-up step was performed, adding OMWW (50% *v*/*v*) into the culture (50% *v*/*v*). All fermentations were carried out at 28 °C for 4 days to obtain alcoholic products, i.e., Aw-Et, Bw-Et, Cw-Et, A-Et, B-Et and C-Et. At the end of the alcoholic fermentation, fermented OMWWs were centrifuged at 6000 rpm at 4 °C, vacuum-filtered and stored at 4 °C until use. The most suitable alcoholic product, to be used as a substrate for the acetification process, was chosen based on the final ethanol and residual sugar content.

### 2.5. Screening of AAB Strains

An AAB screening phase was carried out using *A. pasteurianus* UMCC 1786, *A. pasteurianus* UMCC 1754, *K. europaeus* UMCC 1806, *A. pasteurianus* DSM 3509^T^, and *K. europaeus* DSM 6160^T^. Briefly, an inoculum concentration of 10^8^ CFU/mL of each AAB culture, grown as reported in Section 2.2, was transferred in 100 mL Erlenmeyer flasks containing 30 mL of OMWW previously fermented by *Saccharomyces cerevisiae* UMCC 855. Cultures were incubated for 4 days at 28 °C under static conditions. For each sample, 3 biological replicates were performed. Acetic acid and ethanol content were determined by HPLC (see Section 2.7, the analytical determinations paragraph).

### 2.6. Acetic Acid Fermentations

The acetic acid fermentation was carried out using an *A. pasteurianus* UMCC 1754 strain following the method described by Gullo et al. [22], in the static surface and submerged process. The starter culture of UMCC 1754 was obtained through small-scale batch static fermentation (Phase 1) in 500 mL Erlenmeyer flasks, adding the alcoholic product (B-Et) into the revitalized culture (1:1, *v*/*v*) every 7 days for a total of 28 days at 28 °C.

Phase 2 was conducted in a static regime using 500 mL Erlenmeyer flasks containing P1 and B-Et (1:1, *v*/*v*) at 28°C for 14 days (P2-14). After 7 days, chemical parameters were measured (P2-7), and 3.00 L of P2-7 were added to 3.00 L of B-Et as the starting mash for submerged fermentation (Phase 3). Subsequently, the starting mash was transferred in an 8.0 L fermenter (CETOTEC^®^ GmbH, Germany) operating in semi-batch mode for 14 days at 28°C (P3-14).

After 7 days (P3-7), when the residual ethanol concentration reached 1.90–2.75% (*v*/*v*), 200 mL of the fermented liquid was discharged and replaced with 200 mL of B-Et. Chemical analyses of the samples were performed after 7 (P3-7) and 14 (P3-14) days of fermentation.

### 2.7. Analytical Determinations

The pH of the samples was measured using a pHmeter (MicropH 2002 pHmeter, Crison, Barcellona, Spain), while titratable acidity was determined by titration of the samples with 1 N NaOH up to a pH of 7.0 and expressed as g/100 mL of acetic acid. Sugars, organic acids, and ethanol were measured by HPLC (Jasco LC-Net II/ADC, Germany) equipped with an RI detector (Jasco RI-2031 Plus, Tokyo, Japan) and UV detector (Jasco UV-2070 Plus, Tokyo, Japan) according to Aiello et al. [26]. HPLC standards were purchased from Sigma-Aldrich (Milan, Italy). For the mobile phase, reagents included sulfuric acid 96% from PanReacAppliChem (ITW Reagents, Milan, Italy) and acetonitrile RS for HPLC-GOLD, ultragradient grade, from Carlo Erba Reagents (DasitGroup, Milan, Italy). Briefly, samples were filtered through 0.45 μm PTFE membranes and 20 μL was injected. An isocratic separation of molecules was performed using a Bio-Rad Aminex HPX-87H column (Hercules, CA, USA) (300 × 7.8 mm) heated to 40 °C with an Eldex CH-150 oven. The mobile phase was composed of 0.005 N sulfuric acid and 5% (*v*/*v*) of acetonitrile using a flow of 0.6 mL/min. Peak identifications were conducted using the functions provided by ChromNAV 1.0 software (Jasco, Tokyo, Japan).

### 2.8. Total Phenolic Compounds and Antioxidant Activity

Total phenolic compounds (TPC) were analyzed by the Folin–Ciacolteu method; the results were expressed as mg/L of gallic acid equivalent (GAE) [27].

The radical scavenging activity (RSA) was evaluated using the 2,2-diphenyl-1-picrylhydrazyl (DPPH) assay, following the protocols reported by Palmeri et al. [28]. DPPH was purchased from Sigma-Aldrich (Sintra, Portugal). A methanolic solution of DPPH radical (final concentration 100 µM) was prepared; 3 mL of this solution were mixed with 50 µL of the sample or 50 µL of methanol for the blank. The reaction mixtures were incubated in the dark at 25 °C for 1 h. The absorbance was measured at 515 nm with a spectrophotometer (Jasko V-550, Tokyo, Japan). The RSA was calculated as a percentage using the following equation:

RSA% = [(Absorbance blank − Absorbance sample)/Absorbance blank] × 100

All measurements were performed in triplicate; the results were expressed as the mean percentage of RSA.

### 2.9. Genomic DNA Extraction and Amplification of (GTG)_5_/rep-PCR

Genomic DNA extraction was conducted using the DNeasy^®^ Powerfood^®^ microbial kit GeneElute™ (QIAGEN, Qiagen, Hilden, Germany), according to the manufacturer’s instructions, followed by (GTG)_5_ rep-PCR fingerprinting. gDNA was checked by 1% gel agarose in 1× TBE buffer and quantified by spectrophotometric measurement (NanoDrop ND-1000, Thermo Fisher Scientific, Waltham, MA, USA). Band sizes were determined using GeneRuler 100 bp Plus DNA Ladder (Invitrogen, Carlsbad, CA, USA). (GTG)_5_ rep-PCR fingerprinting was carried out according to the method by Gullo et al. [22]. Strains were subjected to rep-PCR with a single oligonucleotide, GTG_5_ (5′-GTGGTGGTGGTGGTG-3′). Samples were incubated for 5 min at 94 °C and then cycled 35 times at 94 °C for 30 s, 40 °C for 1 min, and 72 °C for 4 min. The samples were incubated for 7 min at 72 °C for final extension and kept at 4 °C. Pattern band lengths were determined by comparison against a 100 bp plus DNA ladder (Takara Bio, Inc., Otsu, Shiga, Japan).

### 2.10. Statistical Analysis

Statistical analysis of the obtained results was performed by one–way analysis of variance (ANOVA) and Tukey’s HSD post hoc test for separation of means at a significance level of *p* ≤ 0.05 and reported as means of the triplicates ± standard deviation. For data processing, SPSS software (version 20.0, IBM Statistics, Armonk, NY, USA) was used.

## 3. Results and Discussion

This study was based on the hypothesis that OMWW, as the main waste product of olive oil production, and having a very high organic load, could be valorized by selective fermentations for producing non-alcoholic fermented beverages containing phenolic compounds. The rationale of the work was to explore selective fermentations operated by yeasts (alcoholic fermentation) and acetic acid bacteria (acetic acid fermentation) of the UMCC culture collection.

### 3.1. OMWW Chemical Characterization and Alcoholic Fermentation

In the literature, OMWW is described as a red-to-black liquid, slightly acidic with a pH of 4.80–5.50, dry matter of 4.12–16.38%, total sugars of 1.5–12.22%, titratable acidity of 0.2%, and total phenols 3830 mg GAE/L [29,30,31]. The physicochemical characteristics of OMWW are variable, depending on the variety of the olives, climatic conditions during growth, and the olive oil extraction method used. In this study, OMWW samples were centrifuged and filtered prior to chemical–physical characterization for assessing their fermentative aptitude and the total phenolic content. The results indicated physicochemical characteristics consistent with previous studies (Table 3).

However, due to the low sugar concentration and the high content of TPC, which could have an inhibitory effect on yeast metabolism [32], OMWW might not be suitable for obtaining alcoholic products with a high enough ethanol content. Indeed, in a previous work, despite the presence of phenols in OMWW, the authors obtained suitable ethanol yields (8–12% *v*/*v*) by adding sugars prior to alcoholic fermentation and balancing the levels of reducing sugars and phenolic fraction [33]. Additionally, as reported by Dourou et al. [34], diluted OMWW was supplemented with carbon and nitrogen sources to improve the final ethanol yield.

**Figure 2:**
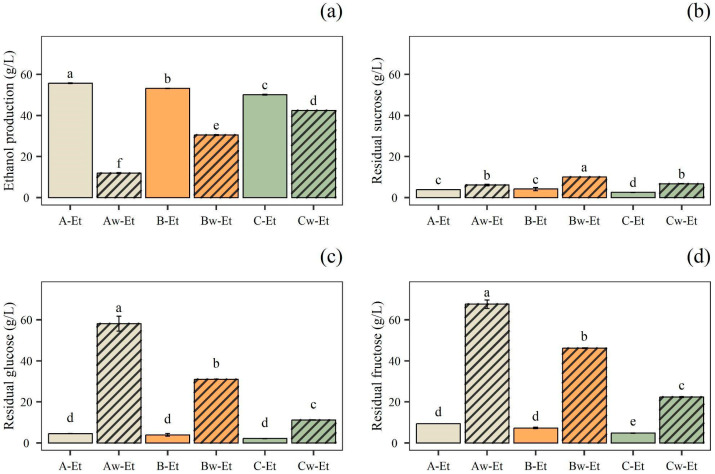
Alcoholic fermentation trials. Quantification of: (**a**) ethanol production. Residual concentration of: (**b**) sucrose; (**c**) glucose; and (**d**) fructose, after alcoholic fermentation of olive mill wastewater with added sucrose (Aw-Et, Bw-Et, Cw-Et) and with added sucrose and nitrogen sources (A-Et, B-Et, C-Et). Data are expressed as mean ± standard deviation (n = 3). Significant differences among analytes are shown by different letters (*p* ≤ 0.05).

In this study, OMWW was diluted at 75% *v*/*v* (B) and 50% *v*/*v* (C) with water to reduce the TPC content and, therefore, the inhibitory effect of the phenolic compounds on microbial cellular metabolism. Undiluted OMWW (A) was used to assess any inhibition caused by the phenolic compounds and the strain’s ability to grow on a stressful substrate. In addition, diluted and undiluted OMWWs were supplemented with sucrose or with sucrose and nitrogen sources. After 4 days of alcoholic fermentation, ethanol production and sugar residual content were determined by HPLC (Figure 2). Among the trials, the residual concentration of sugars was higher in the sucrose-enriched samples (Aw-Et, Bw-Et, and Cw-Et) compared to those containing both sucrose and nitrogen sources (A-Et, B-Et, and C-Et). Consequently, A-Et, B-Et, and C-Et exhibited a higher ethanol content.

Specifically, the sample Aw-Et had the highest residual fructose and glucose content, at 6.77 g/L and 5.82 g/L, respectively, while its ethanol concentration was the lowest among all samples (11.97 g/L). Similarly, Bw-Et and Cw-Et also showed high fructose and glucose residues, along with slightly higher ethanol production, reaching 30.53 g/L and 42.48 g/L, respectively. Conversely, Cw-Et had the lowest residual glucose (11.18 g/L) and fructose (22.41 g/L) among the samples without a nitrogen source, while achieving the highest ethanol content (42.48 g/L).

On the other hand, in samples A-Et, B-Et, and C-Et, the residual fructose was 9.38, 7.31, and 4.83 g/L; the residual glucose was 4.58, 3.95, and 2.17 g/L; and ethanol production reached 55.83, 53.25, and 50.23 g/L, respectively. Thus, our findings suggested that *S. cerevisiae* UMCC 855 performed better in trials supplemented with both sucrose and nitrogen sources than without this latter supplement. As a matter of fact, *S. cerevisiae* requires trace amounts of growth factors, such as vitamins, purines, pyrimidines, nucleotides, nucleosides, amino acids, fatty acids, and sterols, to carry out specific catalytic or structural functions. In alcoholic fermentations, a combination of yeast extract, ammonium phosphate, and minerals can be used to maintain consistent yeast activity [35]. Moreover, ethanol production cannot occur efficiently without substantial yeast cell growth. In support of this, although working on a different substrate than the one used in this work, Laopaiboon et al. [36] reported that the addition of sucrose, yeast extract, and peptone to sweet sorghum juice enhanced sugar consumption, resulting in a higher ethanol concentration and conversion efficiency. In another work, the use of OMWW in the culture media for *S. cerevisiae* increased biomass and ethanol production, highlighting the possible use of OMWW as a promising substrate for the biotechnological production of ethanol [37].

In this study, A-Et exhibited the highest ethanol concentration. However, as the A-Et sample was undiluted, its TPC was considered too high for optimal acetic fermentation. As noted by Pacheco-Ordaz et al. [38], a TPC of 2.70 g/L can drastically inhibit Gram-negative bacteria. Given that AAB are also Gram-negative, this inhibition could negatively impact the acetification process. On the other hand, B-Et had a high ethanol concentration and a low residual sugar content, which is an important feature to consider when developing acetic beverages with health attributes. Furthermore, compared to C-Et, B-Et had a slightly higher ethanol content. Moreover, B-Et presented a lower dilution of the initial sample, i.e., OMWW diluted with distilled water at 75% (*v*/*v*) (B-Et) and 50% (*v*/*v*) (C-Et), which falls within the objectives of maximizing OMWW reuse. To sum up, B-Et was chosen for the subsequential acetification process due to its high ethanol content (53.25 g/L), low residual sugar content, high OMWW initial content, thereby ensuring a greater use of the by-product without having a TPC content that could have compromised the success of the subsequent fermentation phases.

### 3.2. Screening of AAB for the Acetic Acid Fermentations

To develop an efficient acetification process, two *A. pasteurianus* strains, one *K. europaeus* strain, and the respective type strains were screened for their ability to produce acetic acid. In particular *A. pasteurianus* strains are generally used in vinegar production by static systems, whereas *K. europaeus* strains are used in submerged systems [23]. The production of acetic acid, and the ethanol–acetic acid conversion ratio, were set as criteria for selecting the highest-performing strain. In the vinegar fermentation process, initial ethanol concentration represents a stress factor for AAB [39]. Moreover, in the case of OMWW, the presence of phenolic compounds can also represent a hurdle to AAB growth [2]. Hence, during the screening of acetification process, AAB strains were grown in fermented OMWW to evaluate their behavior and ability to produce acetic acid under physiological stress (undiluted OMWW).

**Figure 3:**
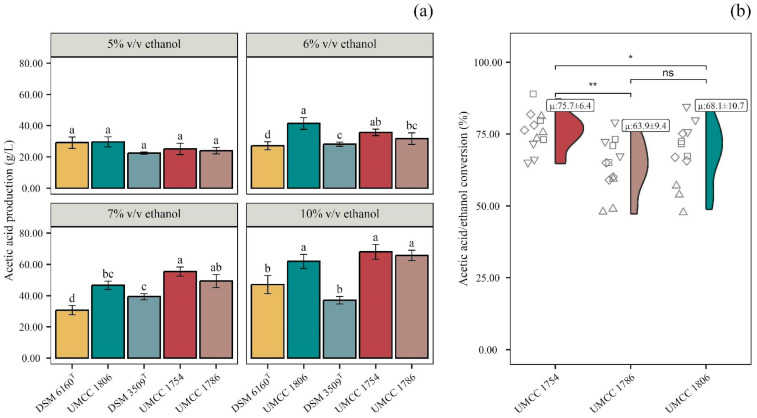
Acetic acid bacteria screening: (**a**) quantification of acetic acid production (g/L) by DSM 6160^T^, UMCC 1806, DSM 3509^T^, UMCC 1754, and UMCC 1786 in OMWW with 5, 6, 7, and 10% (*v*/*v*) of ethanol. Data are expressed as mean ± standard deviation (n = 3). Significant differences among acetic acid production are shown by different letters (*p* ≤ 0.05); and (**b**) overall acetic acid/ethanol conversion ratio among UMCC 1754, UMCC 1786, and UMCC 1806: each point represents the conversion ratio replicate related to the initial ethanol concentration. “μ” indicates the average conversion ratio of the fermented OMWW. Significant difference analysis was performed using the t-test (ns, no significant difference; * *p* ≤ 0.05; ** *p* ≤ 0.01.).

The highest acetic acid producers were *A. pasteurianus* UMCC 1754, *A. pasteurianus* UMCC 1786, and *K. europaeus* UMCC 1806, respectively; no significant differences were observed among all the strains (Figure 3a). For the three highest acetic acid producers, the ethanol–acetic acid conversion ratio was calculated to assess any differences among the strains and highlight the highest-performing one. The conversion ratio was expressed as the percentage of acetic acid produced per amount of ethanol consumed.

As shown in Figure 3b, the averages of the conversion ratios were 75.7, 63.9, and 68.1% for UMCC 1754, UMCC 1786, and UMCC 1806, respectively. The highest value detected was 87.2% by UMCC 1754, which is lower than the theoretical conversion ratio [40]. A partial explanation could be related to the utilization by the cells of the ethanol for the synthesis of cellular constituents and the partial loss by evaporation in the tested conditions. On the other hand, the composition of the substrate is also a key factor, since the presence of positive (e.g., ethanol or nitrogen sources) and negative (e.g., antimicrobial) compounds deeply influences bacterial growth.

Although the samples were diluted to achieve a TPC below 2.70 g/L, which is a threshold for strong microbial inhibition [38], the high phenolic concentrations, along with the presence of compounds exhibiting antimicrobial activity, may have impaired bacterial growth. This effect, potentially due to an extended adaptation phase during the early stages of fermentation, likely contributed to reduced substrate-to-product conversion efficiency [41,42,43].

Overall, UMCC 1786 and UMCC 1806 had a similar conversion ratio, with both strains showing a dependence on the initial ethanol concentration and spread values. On the contrary, UMCC 1754 had a more stable conversion ratio, independently from the initial ethanol concentration, ranging from 64.7 to 87.2%.

Previous studies highlighted the effectiveness of UMCC 1754 in performing the acetic acid fermentation of different raw materials and also the high phenotypic stability after prolonged long-time preservation (9 years) [44]. In particular, UMCC 1754 has been previously tested at the industrial scale for static vinegar production [45]. At the laboratory and prototype scale, it showed a high acetic acid production and a fast start-up phase, highlighting its high suitability and potential as a starter culture for vinegar production [22].

Based on the results of the acetification screening, the strain demonstrating the highest acetic acid yield and ethanol-to-acetic acid conversion efficiency was selected. *A. pasteurianus* UMCC 1754 exhibited the most favorable substrate-to-product conversion ratio and was therefore chosen as the most suitable candidate for acetic beverage production from OMWW.

### 3.3. Acetic Acid Fermentations

**Figure 4:**
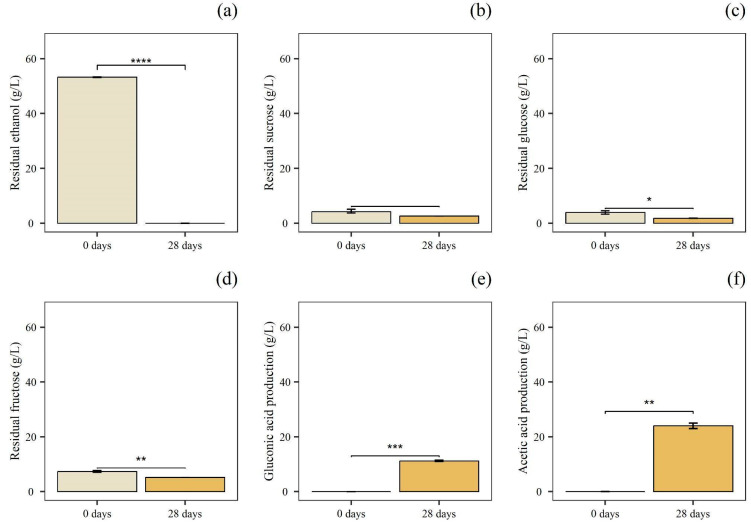
P1, small-scale batch static fermentation. Quantification of residual concentration of: (**a**) ethanol; (**b**) sucrose; (**c**) glucose; and (**d**) fructose. Production of: (**e**) gluconic acid; and (**f**) acetic acid at 0 and 28 days of fermentation. Data are expressed as mean ± standard deviation (n = 3). Significant difference analysis was performed using the t-test (ns, no significant difference; * *p* ≤ 0.05; ** *p* ≤ 0.01; *** *p* ≤ 0.001; **** *p* ≤ 0.0001).

For the acetic acid fermentation, the alcoholic product (B-Et) was inoculated with a culture of *A. pasteurianus* UMCC 1754. To obtain a greater volume of product, as reported in the literature, efficient control of the initiation of acetification and the initial colonization of the substrate by AAB was necessary for the success of the entire bioprocess [46]. Thus, as described by Gullo et al. [22], a scaling-up approach was performed. In detail, during small-scale batch static fermentation, *A. pasteurianus* UMCC 1754 was cultivated for a total of 28 days at 28 °C in B-Et to obtain the starter culture (P1) (Figure 4).

After 28 days, a complete depletion of ethanol and a reduction in glucose concentration were observed. At the same time, acetic and gluconic acid were detected at a concentration of 24.10 g/L and 11.20 g/L, respectively, indicating that the UMCC 1754 strain successfully metabolized both ethanol and glucose.

**Figure 5:**
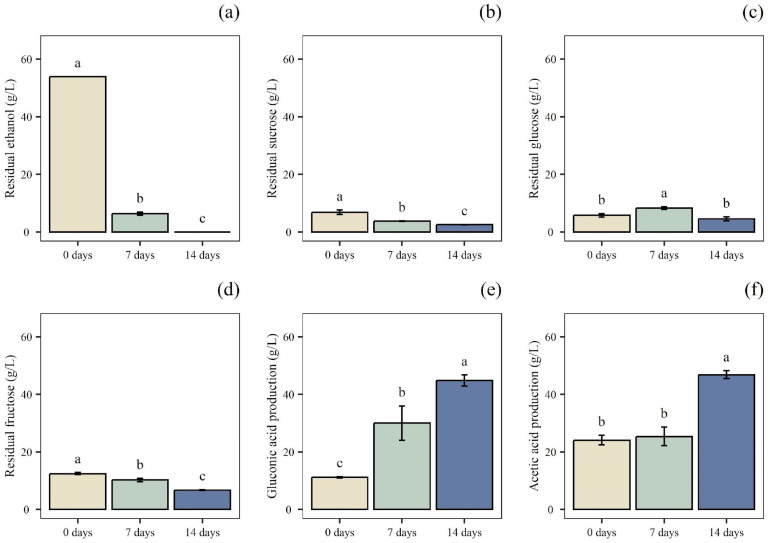
P2, acetic acid fermentation in static regime. Quantification of residual concentration of: (**a**) ethanol; (**b**) sucrose; (**c**) glucose; and (**d**) fructose. Production of: (**e**) gluconic acid; and (**f**) acetic acid at 0, 7, and 14 days of fermentation (Phase 2). Data are expressed as mean ± standard deviation (n = 3). Significant differences among analytes are shown by different letters (*p* ≤ 0.05).

In the acetic product obtained after 14 days of P2 fermentation (P2-14), no residual ethanol was detected, while high amounts of acetic and gluconic acid (46.85 and 44.87 g/L, respectively) were found (Figure 5). Additionally, P2-14 achieved higher values of titratable acidity (4.41%) compared to the sample after 7 days of P2 fermentation (P2-7) (3.90%), along with a decrease in sugar content, demonstrating the continuation of the fermentative process (Table 4).

**Figure 6:**
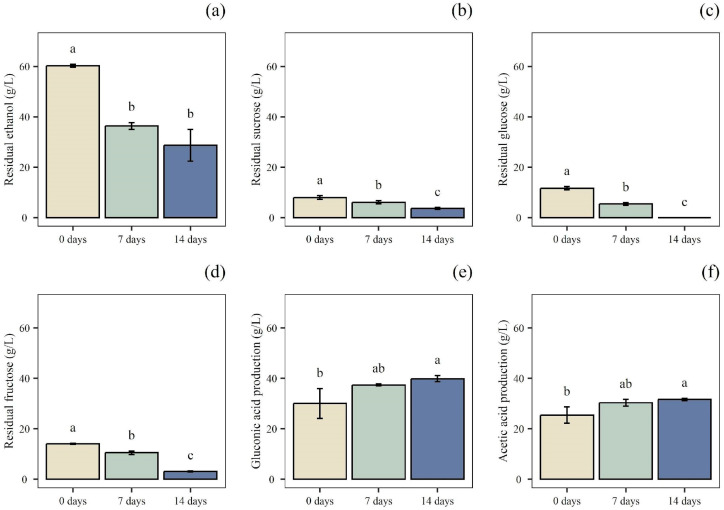
P3, acetic acid fermentation in submerged regime. Quantification of residual concentrations of: (**a**) ethanol; (**b**) sucrose; (**c**) glucose; and (**d**) fructose. Production of: (**e**) gluconic acid; and (**f**) acetic acid, at 0, 7, and 14 days of fermentation in submerged regime (Phase 3). Data are expressed as mean ± standard deviation (n = 3). Significant differences among analytes are shown by different letters (*p* ≤ 0.05).

As reported by others auhtors [25] an ethanol concentration between 5% and 10% positively influences the growth of *Acetobacter* strains. The starting mash of P3 showed an ethanol concentration of 60.33 g/L (Figure 6). These conditions align with the optimal range, supporting the initiation of fermentation and ensuring an adequate substrate supply for bacterial growth and acetic acid production. After 7 days, 200 mL of B-Et was added; the chemical composition after 7 (P3-7) and 14 days (P3-14) is shown in Table 5. During the acetification process in the submerged fermenter, the concentration of ethanol decreased, while acetic and gluconic acid concentration increased, reflecting the enhancement of titratable acidity. Regarding the organic acids content, P3-14 showed a final amount of 31.63 g/L of acetic acid and 39.90 g/L of gluconic acid. On the contrary, no residual amount of glucose was detected. The data obtained are comparable with those detected in the study by Sainz et al. [47]. In fact, strains belonging to the *Acetobacter* genus, during acetic fermentation, are reported to produce both acetic and gluconic acid from ethanol and glucose, respectively [22,48].

Typically, in vinegar production, ethanol is oxidized into acetic acid, leading to an accumulation of this acid in the medium. On the other hand, glucose oxidation is usually observed in sugared substrates, like vinegar produced by cooked must [49], kombucha tea, and other sugary environments leading to gluconic acid production [50,51]. It is known that AAB are obligate aerobic microorganisms and that pyrroloquinoline quinone-dependent glucose dehydrogenase (PQQ-GDH) is the primary enzyme responsible for gluconic acid production [52]. As reported by other authors, an appropriate supply of oxygen is an essential factor in the oxidation reaction involving PQQ-GDH to produce gluconic acid [53,54]. Therefore, the presence of sufficient dissolved oxygen in the growth substrate is positively correlated with an increase in gluconic acid production. This can be explained by the role of the PQQ-GDH system, which involves a major consumption of dissolved oxygen [55]. In this study, the concentrations of organic acids in P2-14 and P3-14 seem to appear similar. Specifically, the concentrations of gluconic acid and acetic acid in P2-14 were 44.87 and 46.85 g/L, respectively, while in P3-14, gluconic acid was 39.90 g/L and acetic acid was 31.65 g/L. However, since Phase 3 employed a 50% (*v*/*v*) inoculum of P2-7, and Phase 2 employed a 50% (*v*/*v*) inoculum of P1, the actual amounts of newly produced gluconic and acetic acids in P3-14 were 9.84 g/L and 6.25 g/L, respectively. Conversely, in P2-14, the newly produced gluconic acid was 33.67 g/L and the acetic acid was 22.82 g/L. These results indicate that the production efficiency of organic acids in Phase 2 was higher than that in Phase 3.

However, the presence of high concentrations of gluconic acid is a valued quality in fermented beverages, so much so that it is commonly added to enhance sensory properties in several products, especially acidic and astringent foods [56].

### 3.4. Total Phenolic Compounds and Antioxidant Activity

**Figure 7:**
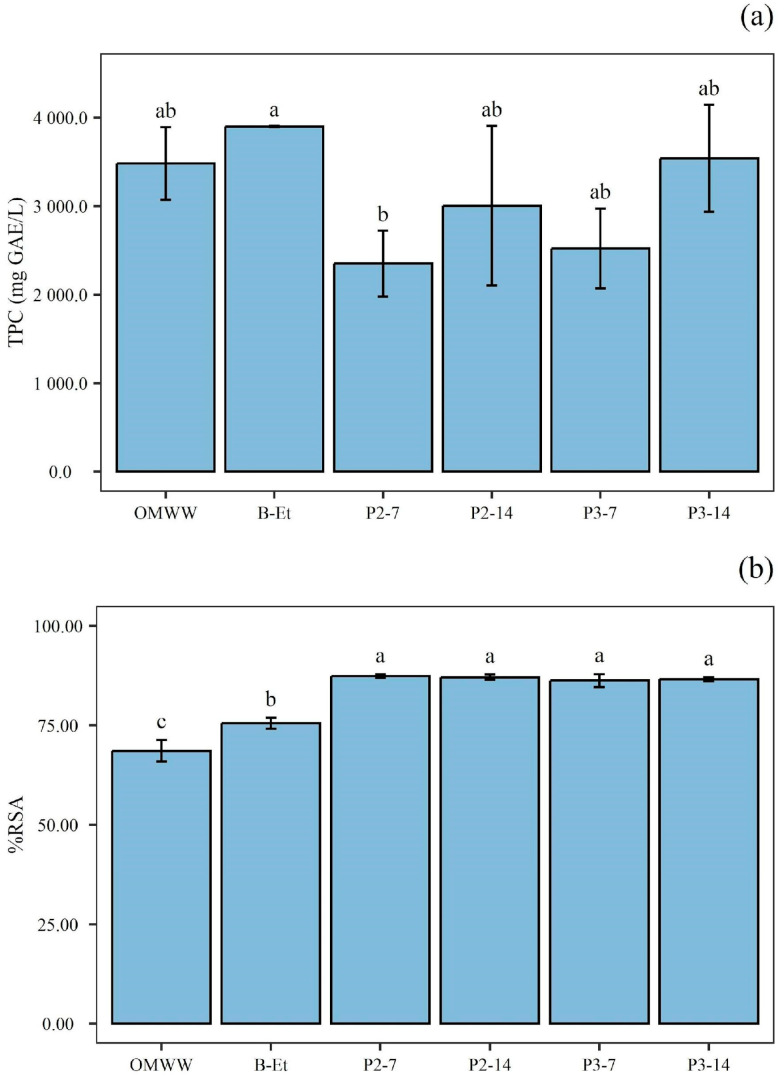
(**a**) Total phenolic compounds (mg GAE/L); and (**b**) antioxidant activity (DPPH assay) of olive mill wastewater (OMWW), OMWW alcoholic product (B-Et), small-scale batch sample after 28 days of fermentation (P1), static fermentation samples after 7 days (P2-7) and 14 days (P2-14), and submerged fermentation samples after 7 days (P3-7) and 14 days (P3-14). Data are expressed as mean ± standard deviation (n = 3). Significant differences among antioxidant activity are shown by different letters (*p* ≤ 0.05).

In this study, along with the acetic acid fermentation time, the phenolic compound concentration increased (Figure 7a) despite an initial decrease compared to OMWW and B-Et. As shown in Figure 7a, the highest TPC values were observed in OMWW, B-Et, and P3-14. This pattern could be attributable to the hydrolysis reactions of oligomeric phenols that occurred during acetic fermentation. Indeed, oligomeric phenols bonded through ester and glucoside linkages [57]. Furthermore, some authors have hypothesized that the increase in TPC may be due to the degradation of phenolic compounds into smaller molecules caused by enzymatic activity during fermentation [58]. Similarly, as reported in several studies on Kombucha tea, the highest TPC values have been observed during the acetic fermentation process [59,60].

The antioxidant activity was evaluated using the DPPH assay. All samples exhibited the ability to scavenge free radicals (Figure 7b), indicating antioxidant activity. The data revealed that OMWW had a lower antioxidant capacity compared to the fermented samples, even though TPC decreased during acetic acid fermentation. Considering the numerous studies showing that fermentation enhances antioxidant activity, this process plays a crucial role in obtaining products with antioxidant properties [61,62]. Specifically, it can be highlighted that the combination of alcoholic fermentation followed by acetic fermentation resulted in a significant increase in the antioxidant activity of the fermented samples compared to the raw OMWW. The results obtained align with the findings of Foti et al. [27], indicating that the OMWW control exhibited a lower antioxidant capacity compared to the fermented samples. These properties position fermented OMWW-based beverages as promising candidates for various applications, offering potential benefits in neutralizing free radicals and oxidative stress, thus protecting cells from damage and reducing the risk of chronic diseases [63]. In particular, the phenolic compounds, such as hydroxytyrosol, flavonoids, and secoiridoid, in olive oil and related products or by-products, have been linked to a lower incidence of cardiovascular disease, atherosclerosis, and colorectal cancer [64].

### 3.5. Testing Survival of A. pasteurianus UMCC 1754 During Static and Submerged Fermentation Phases

The use of the selected culture of AAB for obtaining acetic beverages or vinegars is not a consolidated industrial practice. This is due to a number of constraints widely reported in the literature and is mainly due to difficulties in the cultivation and propagation of AAB strains [65]. In this study *A. pasteurianus* UMCC 1754 was selected for this biotechnological process due to its ease of cultivation, phenotypic stability, and consistent ethanol oxidation to acetic acid after long-term preservation. Moreover, in previous studies the strain did not produce bacterial cellulose in all tested conditions, which is an undesired molecule in vinegar and other acetic beverages [22,43].

Our results confirm the production of acetic acid and gluconic acid, together with the absence of bacterial cellulose, and corroborate the function of UMCC 1754 as a driver of acetic fermentation. To confirm that the process was carried out by the selected strain, a molecular typing method, previously validated on AAB, was used [66].

**Figure 8:**
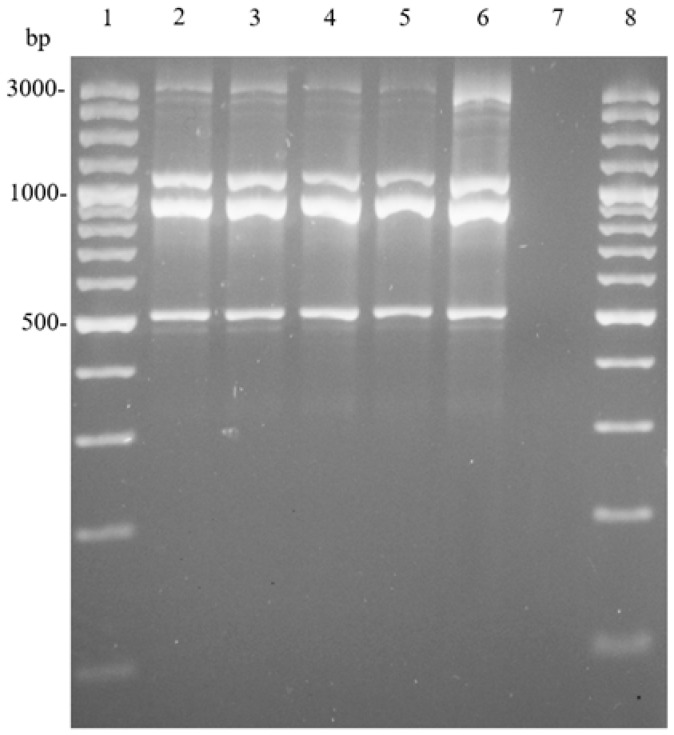
(GTG)_5_-PCR fingerprinting patterns. Line 1: 100 bp Plus DNA Ladder (Thermo Scientific, Carlsbad, CA, USA); line 2: UMCC 1754 in static fermentation after 7 days (P2-7)]; line 3: UMCC 1754 in static fermentation after 14 days (P2-14); line 4: UMCC 1754 in submerged fermentation after 7 days (P3-7); line 5: UMCC 1754 in submerged fermentation after 14 days (P3-14); line 6: UMCC 1754 (*A. pasteurianus*); line 7: negative control; and line 8: GeneRuler 100 bp Plus DNA Ladder.

Samples collected after 7 and 14 days from static fermentation (P2) and submerged fermentation (P3) were analyzed. To ensure the reproducibility of the (GTG)_5_-PCR method, genomic DNA of UMCC 1754 was used as a purity control in each reaction. The size of the amplified DNA fragments ranged from 500 to 3000 bp (Figure 8). The banding patterns of all samples were identical to each other and to that of the original culture strain (Line 6, Figure 8), confirming the occurrence of *A. pasteurianus* UMCC 1754. Moreover, these profiles remained stable in mid-fermentation and persisted until the end of the process. Although several studies have reported that submerged acetification is typically associated with different species of the *Komagataeibacter* and *Novacetimonas* genus [67,68,69,70,71], our results demonstrate that *A. pasteurianus* successfully performed acetic fermentation in all the phases, indicating a strong adaptation to the specific conditions of this process. This suggests its potential for industrial applications.

Based on these preliminary results, the UMCC 1754 strain appears to be a promising candidate as a starter culture, given that gluconic acid and acetic acid are desirable metabolites in acetic beverages. The application of starter cultures is particularly appealing, as it offers multiple advantages, including a reduced fermentation time, a lower risk of spoilage (thus increasing shelf-life), improved process control, an enhanced sensory quality, and safety [72]. Furthermore, the persistence of this strain throughout the entire acetic acid fermentation of OMWW suggests its potential suitability for producing acetic beverages with low or no alcohol at an industrial scale.

## 4. Conclusions

In the Mediterranean region, olive oil production has always been of great economic and cultural significance. However, the olive oil extraction process generates a highly polluting by-product known as OMWW.

The results demonstrated that 75% (*v*/*v*) of OMWW can serve as a suitable substrate for the production of acetic beverages with low or no alcohol content. The presence of carbohydrates supported the hypothesis that the substrate could undergo fermentation by yeasts to produce alcoholic intermediates. However, in this study, in addition to 10% (*w*/*v*) sucrose, 2% (*w*/*v*) yeast extract and 1% (*w*/*v*) bacteriological peptone were also added as nitrogen sources in order to reach a higher ethanol concentration and then, a higher acetic acid amount. A two-step fermentation process—initial alcoholic fermentation by *Saccharomyces cerevisiae*, followed by acetic acid fermentation by *Acetobacter pasteurianus*—proved to be an effective biotechnological approach for OMWW valorization. Two acetic fermentation methods—static and submerged—were applied, both leading to the production of acetic beverages. These findings demonstrate the feasibility of using OMWW in fermentation-based bioprocesses, highlighting its potential to be transformed into value-added beverages. Moreover, the retention of phenolic compounds in the final product enhances the potential health benefits of OMWW-derived acetic beverages, positioning them as functional drinks with potential nutraceutical properties.

In conclusion, this study provides insights into the potential of OMWW as a sustainable raw material for the production of acetic beverages with low or no alcohol, offering an alternative strategy to mitigate the environmental impacts associated with its disposal. However, it is a small-scale and pilot-scale investigation conducted to explore the fermentation capacity and efficiency of OMWW with the selected strains. Obviously, further investigations are needed to apply the bioprocess on a large scale. Surely, operating the scaling-up is a methodology that, on the one hand, is necessary for the adaptation of AAB. On the other hand, it could also facilitate the adaptability of the process in the industry using larger working volumes. In addition, the choices made to use the lowest water content for sample dilution and also for the pre-treatment step of the substrate by filtration were focused on the perspective of the greater economic feasibility of the entire process. Moreover, the addition of sucrose, useful to increase the yield of microorganisms in ethanol production, could be replaced with other substrates of sugar-processing waste, increasing the cost effectiveness in industrial realities.

Further studies will be carried out to properly, and more extensively, implement prototype-scale experiments and evaluate the features and the life cycle of the new product. Furthermore, it will be crucial to understand the variation of the phenolic compounds present at the beginning and end of the process, assessing through specific techniques, like mass spectrometry, the acetic beverage’s phenolic composition for potential beneficial health claims.

## Figures and Tables

**Table 1:** Strains used in this study.

Strain	Species	Isolation Source
UMCC 855 = 21T2	*Saccharomyces cerevisiae*	Wine [17]
DSM 6160^T^	*Komagataeibacter europaeus*	Vinegar [23]
UMCC 1806 = ZJ555	*Komagataeibacter europaeus*	Cereal vinegar [24]
DSM 3509^T^	*Acetobacter pasteurianus*	Beer (Leibniz Institute DSMZ)
UMCC 1754 = AB0220	*Acetobacter pasteurianus*	Wine vinegar [25]
UMCC 1786 = DL13	*Acetobacter pasteurianus*	Cereal vinegar [24]

**Table 2:** Sample codification of acetic acid fermentations.

Process	Time (Days)	Code
Small scale batch static fermentation, Phase 1	28	P1
Static fermentation, Phase 2	7	P2-7
	14	P2-14
Submerged fermentation, Phase 3	7	P3-7
	14	P3-14

**Table 3:** Physicochemical characteristics of olive mill wastewater (OMWW).

Sample	pH	Glucose (g/L)	Fructose (g/L)	Titratable Acidity (% *w*/*v*)	TPC (mg GAE/L)	Dry Matter (% *w*/*v*)
OMWW	4.99 ± 0.07	24.91 ± 0.93	24.11 ± 0.91	0.24 ± 0.01	3480.60 ± 409.12	5.60 ± 0.02

Data are expressed as mean ± standard deviation (n = 3).

**Table 4:** Chemical parameters detected in static condition.

Time (Days)	pH	Titratable Acidity (% *w*/*v*)
0	3.47 ± 0.16 ^a^	1.80 ± 0.20 ^c^
7	3.69 ± 0.02 ^a^	3.90 ± 0.13 ^b^
14	3.70 ± 0.03 ^a^	4.41 ± 0.05 ^a^

Data are expressed as mean ± standard deviation (n = 3). Data within the same column with different letters are significantly different (*p* ≤ 0.05).

**Table 5:** Chemical parameters detected in submerged fermentation.

Time (Days)	pH	Titratable Acidity (% *w*/*v*)
0	4.23 ± 0.84 ^a^	2.29 ± 0.17 ^c^
7	4.14 ± 0.10 ^a^	2.56 ± 0.01 ^b^
14	4.08 ± 0.27 ^a^	2.91 ± 0.03 ^a^

Data are expressed as mean ± standard deviation (n = 3). Data within the same column with different letters are significantly different (*p* ≤ 0.05).

## Data Availability

The original contributions presented in the study are included in the article, further inquiries can be directed to the corresponding author.
